# Shape-based reconstruction of dynamic fluorescent yield with a level set method

**DOI:** 10.1186/s12938-016-0124-y

**Published:** 2016-01-14

**Authors:** Xuanxuan Zhang, Jiulou Zhang, Jing Bai, Jianwen Luo

**Affiliations:** Department of Biomedical Engineering, School of Medicine, Tsinghua University, Beijing, 100084 China; Center for Biomedical Imaging Research, School of Medicine, Tsinghua University, Beijing, 100084 China

**Keywords:** Fluorescence molecular tomography, Dynamic, Level set method, Shape-based reconstruction

## Abstract

**Background:**

Fluorescence molecular tomography (FMT) is an optical imaging technique that reveals biological processes within small animals through non-invasively reconstructing the distributions of fluorescent agents. The primary problem in FMT with non-stationary fluorescent yield is the increase of the unknown parameters to be reconstructed. In this paper, a method is proposed to reconstruct dynamic fluorescent yield.

**Methods:**

A shape-based reconstruction method that recovers dynamic fluorescent yield with a level set method is proposed for FMT. To reduce the number of unknown parameters, a level set function is introduced to describe the shape of target and a small number of parameters are used to describe the fluorescent yields at different time points.

**Results:**

Results of simulations and phantom experiments demonstrate that the proposed method can recover well the dynamic fluorescent yields, shapes and locations of the target.

**Conclusions:**

The proposed method can handle the cases with non-stationary fluorescent yields and recover the fluorescent yields at each projection angle.

## Background

Fluorescence molecular tomography (FMT) [1] is a promising imaging technique that reveals the distributions of fluorescent agents within small animals in vivo. As an optical tomographic imaging technique, FMT has advantages of low cost, non-ionizing radiation and high sensitivity [2]. These merits lead to the popularity of the studies of FMT that not only observe static distribution of fluorescent markers [3] but also investigate metabolic process of drugs [4]. Compared with the static distribution, metabolic characteristics can provide additional information, which is important for drug development and tumor studies. At present, two kinds of methods are used in the reconstruction of dynamic FMT. The first one is to directly apply the reconstruction methods for static FMT to deal with dynamic cases [4, 5]. However, these methods are incapable of recovering the metabolic information within the data acquisition time and perform poorly especially when the fluorophore concentrations vary rapidly [6], because they only reconstruct a static distribution of fluorescent yield from data sets acquired within several minutes. The other kind of methods is based on different models. These methods model the metabolic process with a two-compartmental model [7, 8] or a polygonal line model [9, 10], and reconstruct the distributions of four pharmacokinetic parameters in the two-compartmental model or the distributions of fluorophore concentration variation rate, instead of the distribution of fluorescent yield. These model-based methods convert the inverse problem of dynamic FMT from reconstructing a series of static distributions of fluorescent yield to reconstructing the distributions of other parameters that can represent the metabolic information. Their limitations are the restriction of the used model. In this paper, a new approach is proposed to reconstruct dynamic fluorescent yield based on a level set method. Different from the model-based methods, this method still reconstructs the distributions of fluorescent yield, thus the target metabolic curves are not restricted by specific models. This method is capable of recovering the distributions of fluorescent yield at each projection angle.

If the fluorescent yield is considered as the unknown parameter, the primary problem in FMT reconstruction with dynamic fluorescent yield is that the number of unknown parameters to be reconstructed is dramatically increased, because the fluorescent yield varies projection by projection in the dynamic problem whereas it does not change in the static problem. If the distributions of fluorescent yield at each projection angle are considered to be reconstructed, the number of unknown parameters will be multiplied by the number of projection angles (*N*_*p*_). Based on prior information that the same tissue or organ should have similar metabolic property, a shape-based reconstruction strategy is implemented in FMT to reduce the number of unknown parameters. In this strategy, the reconstructed object is divided into numbers of disjoint subregions and the shapes of these subregions are represented by a level set method [11] or spherical harmonics expansion [12]. Accordingly, the fluorescent yield in each subregion is described by only one parameter. It changes the reconstruction problem from reconstruction of fluorescent yield at each voxel or node to reconstruction of level set function or expansion coefficients with several parameters of fluorescent yield. When a problem with dynamic fluorescent yield is considered, the number of unknown parameters will be reduced because the shapes of the distributions of fluorescent yield at different projection angles are described by the same set of unknown parameters by using the level set method or spherical harmonics expansion. In this paper, the level set method is chosen to represent the shapes of subregions for its ability of arbitrary boundary representation. Although spherical harmonics expansion can also describe arbitrary boundary theoretically, the maximum degree of spherical harmonics, which controls the shape representation, needs to be determined manually [13].

## Methods

The generation and propagation of fluorescence in continuous wave FMT can be described by a couple of diffusion equations with Robin-type boundary condition, which are given by [14, 15]:

1 $$\left\{ {\begin{array}{*{20}c} { - \nabla D\nabla {\rm\phi}_{x} (r) + \mu_{a} {\rm\phi}_{x} (r) = Q(r)} \\ { - \nabla D\nabla {\rm\phi}_{m} (r) + \mu_{a} {\rm\phi}_{m} (r) = {\rm\phi}_{x} (r)\eta \mu_{af} (r)} \\ \end{array} } \right.r \in \Omega$$

2 $$2qD\frac{\partial {\rm\phi} (r)}{{\partial \vec{n}}} + {\rm\phi} (r) = 0\begin{array}{*{20}c} {} & {r \in \partial \Omega } \\ \end{array}$$

where Φ is the photon density. *r* denotes the coordinates and Ω denotes the region of imaged object. The subscripts *x* and *m* represent excitation and emission, respectively. *Q* is the excitation source term. $\vec{n}$ is the outward normal vector to the surface and *q* is a term related to the optical reflective index mismatch at the boundary. *μ*_*a*_ and *D* are the absorption and diffusion coefficients, respectively. *η* is the quantum yield and *μ*_*af*_ is the absorption coefficient of fluorophore to the excitation light. Their product is the fluorescent yield which needs to be reconstructed.

Numerical approaches are commonly used to solve the diffusion equations with arbitrary shaped domains. In this work, finite element method (FEM) is chosen to solve the forward problem, which converts the coupled diffusion equations given by Eq. (1) into the following linear equations after discretizing the imaged domain into a mesh with *N*_*n*_ nodes [16]:

3 $$\left\{ {\begin{array}{*{20}c} {K{\rm\phi}_{x} } &=& Q \\ {K{\rm\phi}_{m} } &=& {FX} \\ \end{array} } \right.$$

Here Φ, *X*, and *Q* are column vectors with a length of *N*_*n*_∙ *X* = *ημ*_*af*_ is the vector of the unknown fluorescent yield. *K* is an *N*_*n*_ × *N*_*n*_ matrix which is commonly called stiffness matrix in FEM. *F* is a matrix with the same size as *K*, which is related to the excitation light field. *K* and *F* are constructed according to the topological structure of the mesh. *K*, *F*, and *Q* are given by:

4 $$K_{i,j} = \int\limits_{\Omega } {(D\nabla {\rm\phi}_{i} \cdot \nabla {\rm\phi}_{j} + \mu_{a} {\rm\phi}_{i} {\rm\phi}_{j} )\;dr^{n} } + \frac{1}{2q}\int\limits_{\partial \Omega } {{\rm\phi}_{i} {\rm\phi}_{j} dr^{n - 1} }$$

5 $$F_{i,j} = \int\limits_{\Omega } {{\rm\phi}_{x} (r){\rm\phi}_{i} {\rm\phi}_{j} dr^{n} }$$

6 $$Q_{i} = \int\limits_{\Omega } {Q(r){\rm\phi}_{i} dr^{n} }$$

where the subscripts *i* and *j* denote the indices of column and row, respectively. *φ*_*i*_ is the shape function at node *i*.

To reduce the number of unknown parameters, the shape-based reconstruction strategy is used. The imaged domain is assumed to consist of several disjoint subregions and the fluorescent yield in each subregion is represented by a single parameter. For simplicity, the case with two subregions (target region *R*_*f*_ and background region *R*_*b*_) is considered here and *x*_*f*_ and *x*_*b*_ are used to denote the corresponding fluorescent yields. To describe the shape of the subregions, a level set function is introduced [17]:

7 $$x\left( {r,t} \right) = \left\{ {\begin{array}{*{20}c} {\begin{array}{*{20}c} {x_{f} \left( t \right)} & {\psi \left( r \right) \le 0} \\ \end{array} } \\ {\begin{array}{*{20}c} {x_{b} \left( t \right)} & {\psi \left( r \right) > 0} \\ \end{array} } \\ \end{array} } \right.$$

where *ψ* is the level set function. Because the case with dynamic fluorescent yield is considered, *x*, *x*_*f*_, and *x*_*b*_ are functions of time.

In order to combine the level set function with FEM, *ψ* is discretized into an *N*_*n*_ dimensional vector Ψ of basis coefficients by a basis expansion with the shape functions as follows:

8 $$\psi \left( r \right) = \sum\limits_{i = 1}^{{N_{n} }} {\psi_{i} {\rm\phi}_{i} }$$

where *ψ*_*i*_ is the level set function at node *i*.

Then Eq. (7) is reformed by a single equation as follows:

9 $$X\left( t \right) = x_{b} \left( t \right)H\left( \Psi \right) + x_{f} \left( t \right)\left[ {1 - H\left( \Psi \right)} \right]$$

where *H* (*ψ*) is the step function which is equal to 0 or 1 if *ψ* ≤ 0 or *ψ* > 0.

Through Eqs. (8) and (9), the unknown parameters to be reconstructed are converted from numbers of vectors *X* into only one vector *Ψ* with a set of values of fluorescent yields {*x*_*f*_ (*i*)} and {*x*_*b*_ (*i*)} after the discretization of the time *t*.

To reconstruct Ψ, {*x*_*f*_ (*i*)} and {*x*_*b*_(*i*)} from boundary measurements, the following object function is defined:

10 $$\Gamma = \frac{1}{2}\left\| {y^{*} - y} \right\|^{2} = \frac{1}{2}\sum\limits_{k = 1}^{{N_{f} }} {\sum\limits_{i = 1}^{{N_{p} }} {\sum\limits_{j = 1}^{{N_{d} }} {\left( {P_{ij} K^{ - 1} F_{i} X_{k,i} - y_{k,ij} } \right)^{2} } } }$$

where *P* is the measurement operation vector [18] used to extract the photon density at a certain detector point from the vector Φ. *N*_*f*_ denotes the number of frames of data used in the reconstruction and *N*_*d*_ denotes the number of detector points at each projection angle. y^*^ and *y* are the predicted measurements calculated from the forward problem and measurements acquired by the system, respectively.

Differentiating Eq. (10) with respect to *ψ*_*m*_ yields:

11 $$\frac{\partial \Gamma }{{\partial \psi_{m} }} = \sum\limits_{k = 1}^{{N_{f} }} {\sum\limits_{i = 1}^{{N_{p} }} {\sum\limits_{j = 1}^{{N_{d} }} {\left( {y_{k,ij}^{*} - y_{k,ij} } \right)P_{ij} A_{i} \left( m \right)} } } \left[ {x_{b} \left( {k,i} \right) - x_{f} \left( {k,i} \right)} \right]\delta \left( {\psi_{m} } \right)$$

where *A*_*i*_ (*m*) denotes the *m*th column of matrix *K*^−1^*F*_*i*_∙ *δ* (*ψ*_*m*_) is the Dirac function which is the derivation of the step function. *x*_*b*_ (*k, i*) and *x*_*f*_ (*k, i*) denote the fluorescent yield at the time point of the *k*th frame and *i*th projection angle for the background and fluorescent target, respectively.

Accordingly, differentiating Eq. (10) with respect to *x*_*f*_ (*k, i*) yields:

12 $$\frac{\partial \Gamma }{{\partial x_{f} \left( {k,i} \right)}} = \sum\limits_{j = 1}^{{N_{d} }} {\left( {y_{k,ij}^{*} - y_{k,ij} } \right)\sum\limits_{{n \in R_{f} }} {P_{ij} A_{i} \left( n \right)} }$$

In Eq. (12), the indices of the nodes in *R*_*f*_ are needed, which are found with Ψ ≤ 0. The derivation with respect to *x*_*b*_ (*k, i*) is similar to Eq. (12) whereas *R*_*f*_ is replaced by *R*_*b*_.

The gradient of the object function consists of {∂Γ/∂*ψ*_*m*_} and {∂Γ/∂*x*_*f*|*b*_ (*k*, *i*)}. The gradient descent method [19] is used to reconstruct {*x*_*b*_ (*k, i*)}, {*x*_*f*_ (*k, i*)} and Ψ from measurements:

13 $$\Psi^{{\left( {n + 1} \right)}} = \Psi^{\left( n \right)} - \tau_{1} \sum\limits_{k = 1}^{{N_{f} }} {J^{T} \left[ {\begin{array}{*{20}c} {\left( {x_{b} \left( {k,1} \right) - x_{f} \left( {k,1} \right)} \right)\left( {Y_{k,1}^{*} - Y_{k,1} } \right)} \\ \vdots \\ {\left( {x_{b} \left( {k,N_{p} } \right) - x_{f} \left( {k,N_{p} } \right)} \right)\left( {Y_{{k,N_{p} }}^{*} - Y_{{k,N_{p} }} } \right)} \\ \end{array} } \right]}$$

14 $$x_{f|b}^{{\left( {n + 1} \right)}} (k,i) = x_{f|b}^{\left( n \right)} (k,i) - \tau_{2} \sum\limits_{{k \in R_{f|b} }} {J_{i}^{T} \left( k \right)\left( {Y_{k,i}^{*} - Y_{k,i} } \right)}$$

where the superscripts (*n*) and (*n* + 1) denote the indices of iteration. *τ*_1_ and *τ*_2_ are the step lengths. They are determined empirically according to the value of the cost function. *Y*_*k, i*_ is the vector of measurements acquired at the *k*th frame and *i*th projection angle. *J* is the Jacobian matrix of the forward operator, which consists of row vectors {*P*_*ij*_*K*^−*1*^*F*_*i*_}. Because the Dirac function in Eq. (11) cannot be numerically calculated, it is omitted in the reconstruction. During the reconstruction, a low-pass filter [20] is applied to the reconstructed fluorescent yields after each iteration to smooth the curve of the reconstructed fluorescent yields.

## Results and discussion

**Fig. 1:**
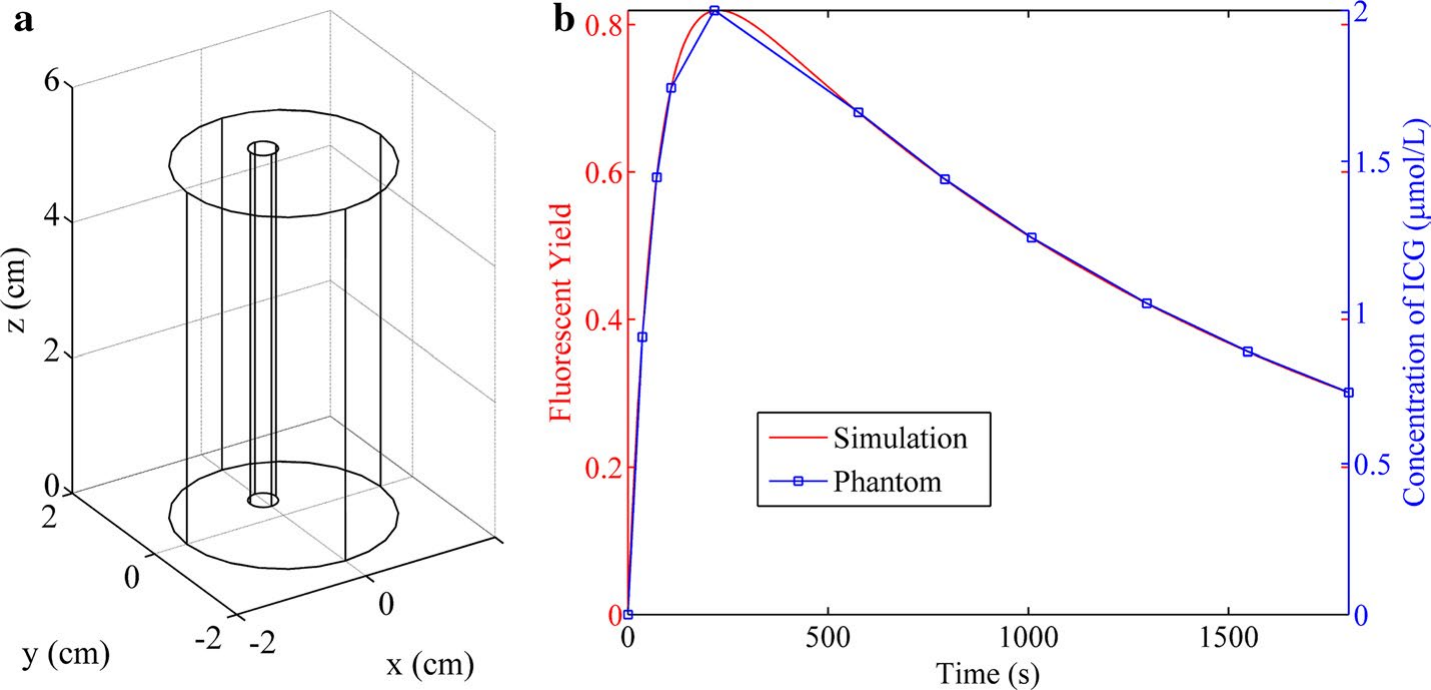
Experimental setup for the studies of single target. **a** The geometry of the model used in the simulation and phantom studies. **b** The curves of fluorescent yield (*red*) or ICG concentration (*blue*) used in the simulation or phantom studies

Simulation and phantom studies were implemented to validate the performance of the proposed method. The experimental setup of the simulation and phantom studies is shown in Fig. 1. The imaged object was a cylinder with a diameter of 3 cm and height of 5.2 cm as shown in Fig. 1a. A tube with a diameter of 0.4 cm and height of 5.2 cm was inserted in the cylinder as the target. The tube was located at the position (*x*, *y*) = (0, 0.5) cm. In the simulations, a varied fluorescent yield according to the red curve shown in Fig. 1b was assigned to the tube. The curve was generated with a metabolic model of indocyanine green (ICG) [21]. Measurements were generated by Eq. (3) and 1 % Gaussian noise was added. In the phantom experiments, ten sets of measurements were acquired when different concentrations of ICG according to the blue points in Fig. 1b were filled in the tube. Then the dynamic measurements were synthesized by linear interpolation.

**Fig. 2:**
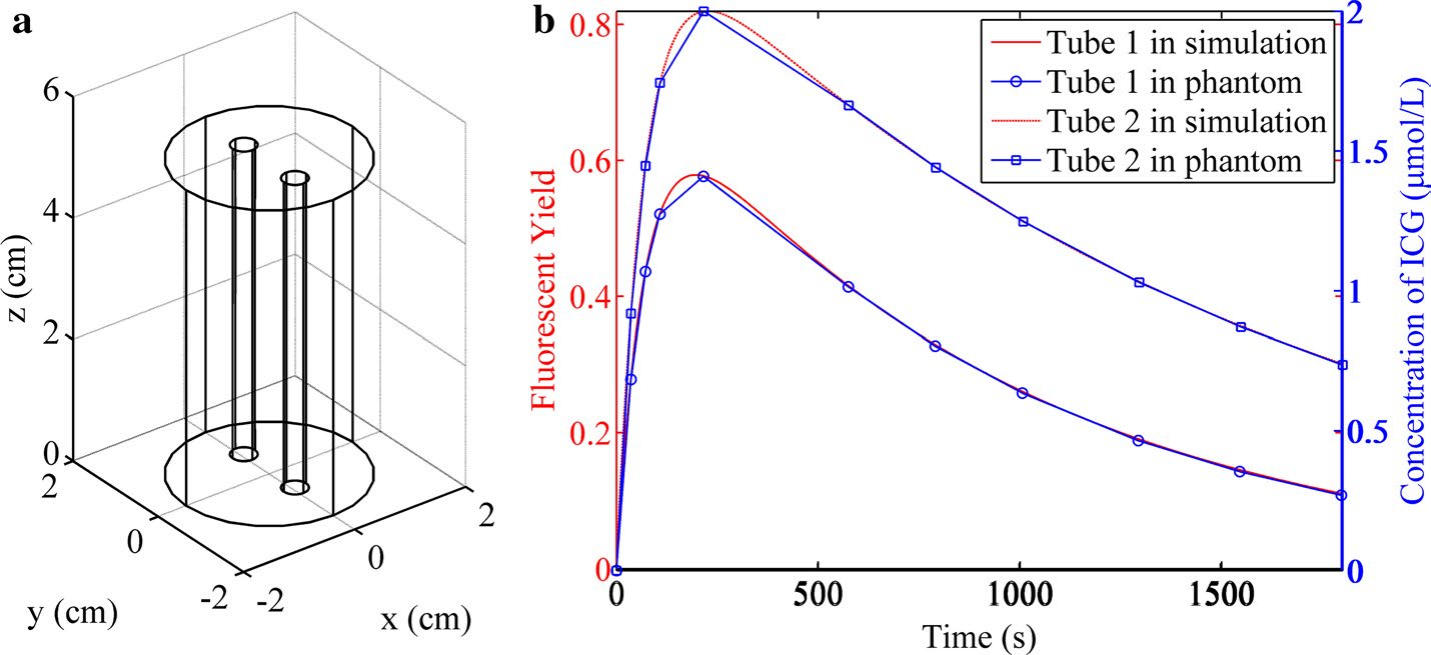
Experimental setup for the studies of double targets. **a** The geometry of the model used in the simulation and phantom studies. **b** The curves of fluorescent yield (*red*) or ICG concentration (*blue*) used in the simulation or phantom studies

Simulations and phantom experiments on a model with double targets as shown in Fig. 2a were also implemented. Two tubes were inserted in the cylinder as the fluorescent targets and the fluorescent yields or concentrations of ICG were set according to two different curves as shown in Fig. 2b. The heights and radii of the cylinder and tubes were the same as those in the previous experimental setting. The two tubes were away from each other with an edge-to-edge distance of 0.8 cm.

**Fig. 3:**
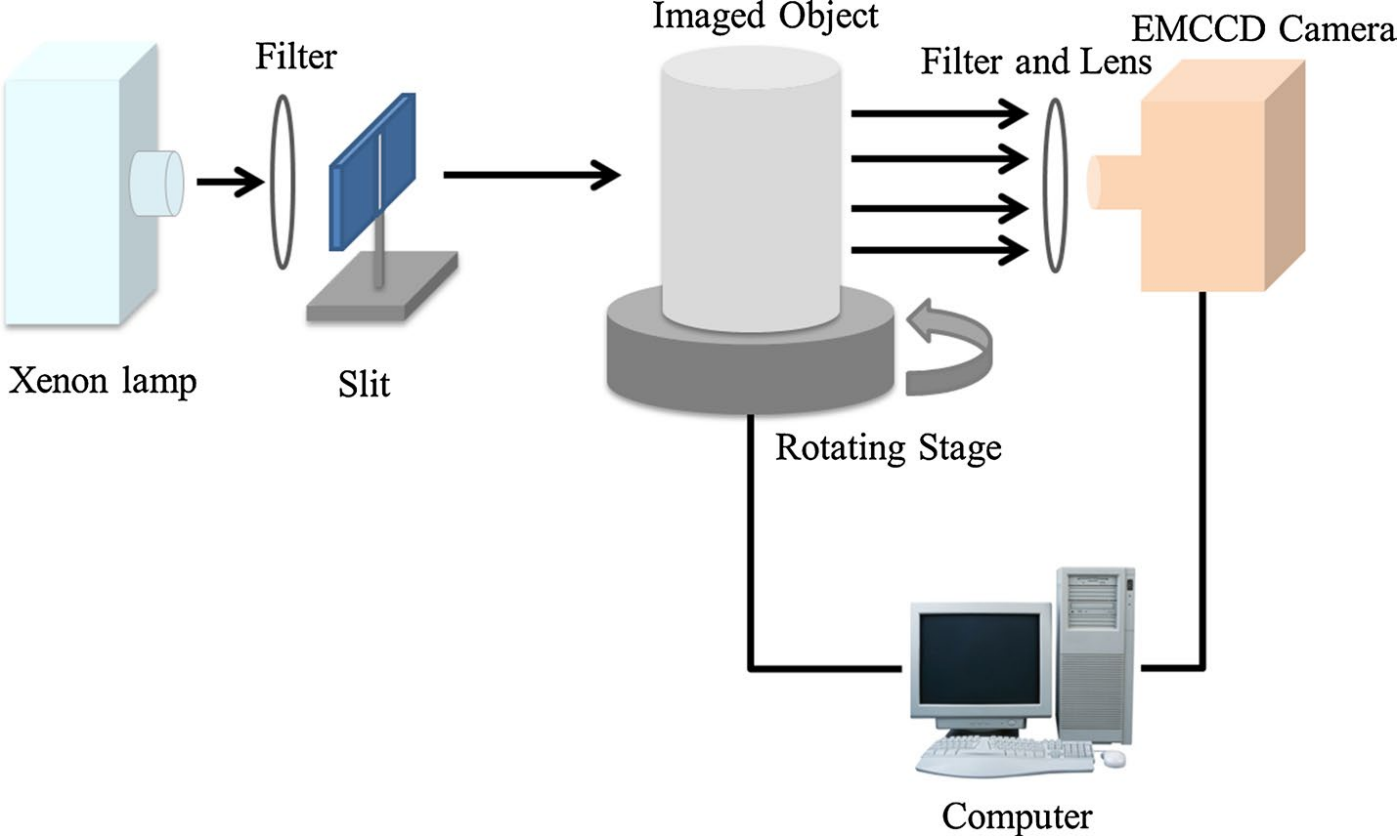
Schematic of the parallel excitation based FMT system

In the phantom experiments, 1 % intralipid with *μ*_*s*_^′^ = 10.0 cm^−1^ and *μ*_*a*_ = 0.02 cm^−1^ was filled in the transparent cylinder. Accordingly, the same optical coefficients were used in the simulations. A parallel excitation based FMT system [22] as shown in Fig. 3 was used to acquire datasets in the experiments. The system consisted of three parts: excitation module, rotation stage, and detection module. The excitation module was a 300 W xenon lamp (Asahi Spectra, Torrance, CA, USA) coupled with a 770 ± 6 nm bandpass filter. A slit was used to generate a line illumination as the excitation source. The detection module consisted of a −70º cooled electron multiplying charge-coupled device (EMCCD) camera (iXon DU-897, Andor Technologies, Belfast, Northern Ireland, U. K.), a Nikkor 60 mm, f/2.8D lens (Nikon, Melville, NY, USA), and an 840 ± 6 nm bandpass filter. The phantom was placed on the rotation stage between the excitation and detection modules and measurements were acquired at 18 projection angles over 360º illuminations and detections. The time for the data acquisition of each projection angle was 2 s. The same time interval was used in the simulations. After the detection of fluorescence, 72 white light images were acquired to recover the geometry of the imaged object with a surface reconstruction method [23].

In the reconstructions, the initial level set functions Ψ^(0)^ were set as a small positive constant for every node, but the value (i.e., 1) used in the phantom studies was chosen to be larger than the one (i.e., 0.001) used in the simulations because the data acquired by EMCCD and the data used in the simulations had different orders of magnitude. Since the level set functions were set to be homogenous at the beginning, the initial subregions consisted of only the background. The initial values of the fluorescent yield *x*_*b*_ were set as 0 for all time points. It should be noticed that *x*_*f*_ and *x*_*b*_ cannot be the same, otherwise the gradient given by Eq. (13) would become 0. Therefore, the initial values of *x*_*f*_ at each time point were set as 1000 and 0.1 for the phantom and simulation studies, respectively.

**Fig. 4:**
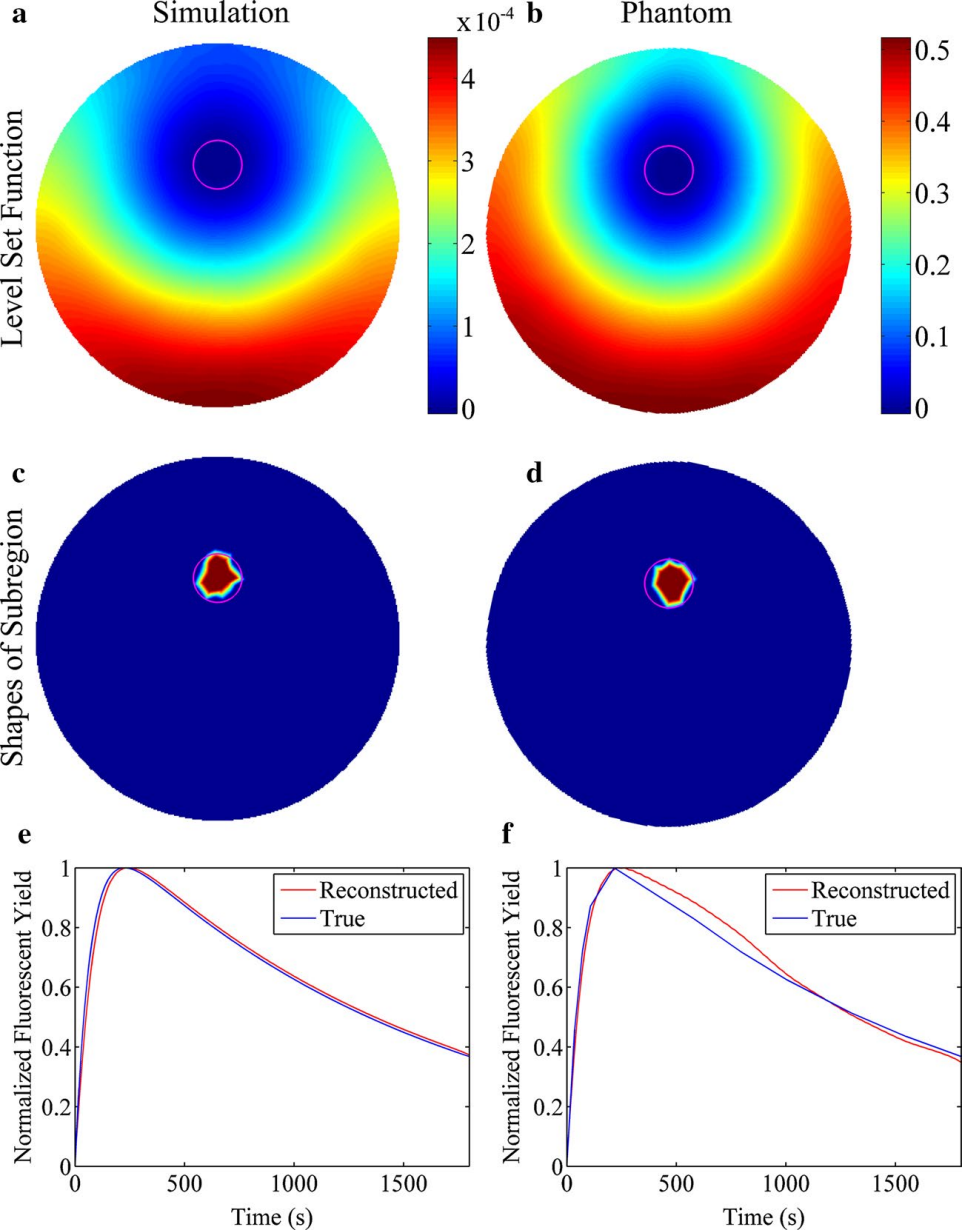
Reconstruction results at the center slice for the studies of single target. The *first* and *second columns* show the reconstruction results in the simulation and phantom studies, respectively. **a** and **b** The reconstructed level set functions. **c** and **d** The shapes of the subregions restricted by the reconstructed level set functions. **e** and **f** The reconstructed fluorescent yields as a function of time

**Fig. 5:**
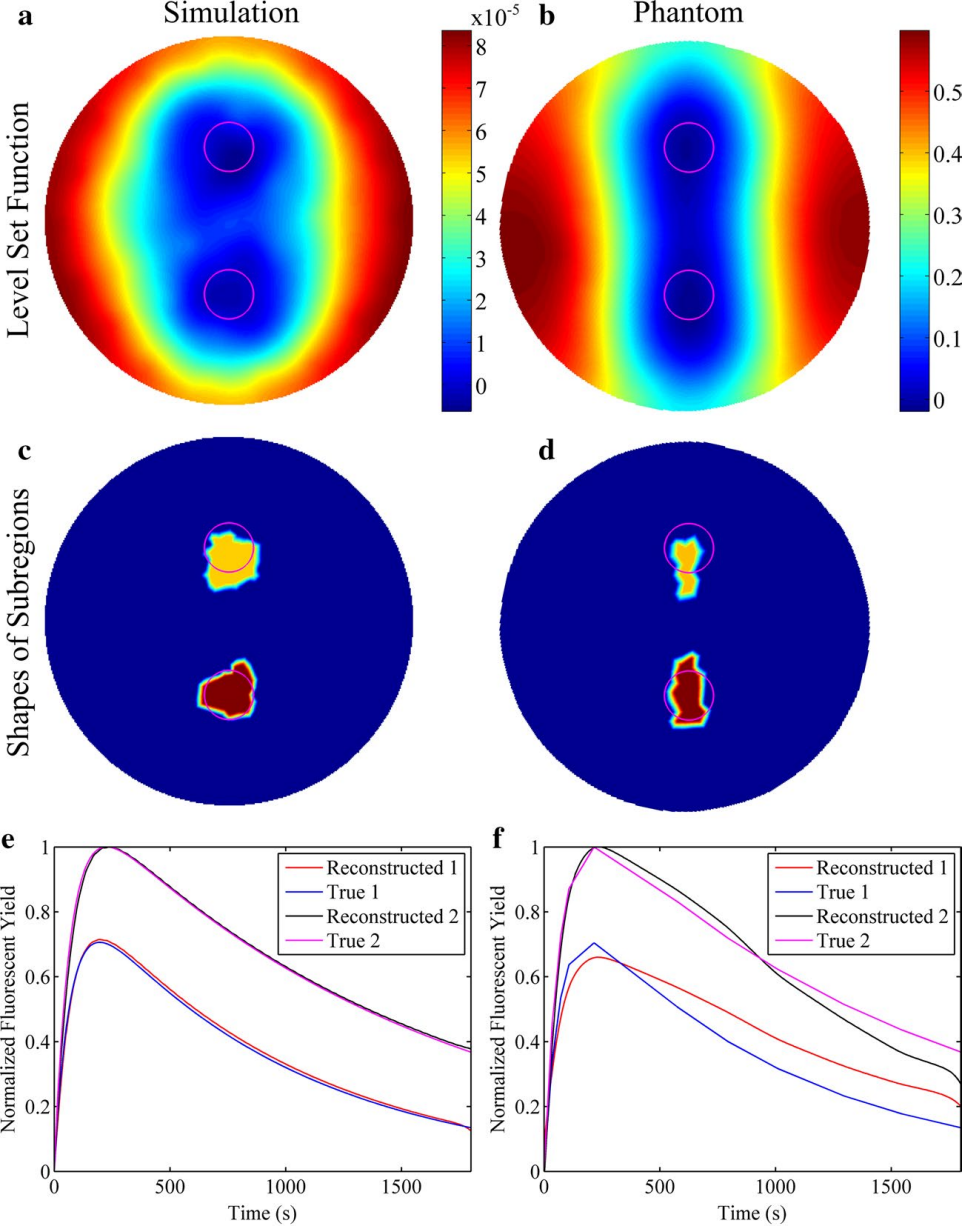
Reconstruction results at the center slice for the studies of double targets. The *first* and *second columns* show the reconstruction results in the simulation and phantom studies, respectively. **a** and **b** The reconstructed level set functions. **c** and **d** The shapes of the subregions restricted by the reconstructed level set functions. **e** and **f** The reconstructed fluorescent yields as a function of time

The reconstructions were restricted within a plane instead of the whole geometry for simplicity, due to the use of the line illumination, cylindrical phantom and tube. The reconstruction results at the center slice are shown in Figs. 4 and 5. The first and second columns of Figs. 4 and 5 are the results of the numerical simulations and phantom experiments, respectively. The subfigures (a) and (b) of Figs. 4 and 5 are the distributions of the level set functions reconstructed by the proposed method, while the subfigures (c) and (d) of Figs. 4 and 5 are the corresponding shapes of the subregions restricted by Ψ ≤ 0. The reconstructed fluorescent yields normalized by the maximum are shown in the subfigures (e) and (f) of Figs. 4 and 5 as time courses. The time interval between two neighbor fluorescent yields is equal to the time for the data acquisition of each projection angle (2 s). It can be found that the reconstructions of both simulations and phantom experiments recover well the dynamic fluorescent yields, shapes and locations of the target. The reconstructed time course for the phantom experiments shows greater mismatch with the true one than that for the simulations, which is probably due to the complicated noise in the experimental datasets of different frames.

In this paper, the iteration formulas given by Eqs. (13) and (14) are derived from the gradient descent method. These formulas are similar to those derived from an artificial time evolution approach [11, 17, 24]. The Dirac function in the gradient for the level set function is omitted in both the proposed method and the methods based on the artificial time evolution approach. Rigorously, Eq. (13) is only proper for those locations with *ψ* = 0, because *δ* (*ψ*) is not zero only when *ψ* = 0. However, the gradient given by Eq. (13) shows the capability of finding the subregion of the target by decreasing the level set function with positive values until it becomes negative in numerical applications.

## Conclusion

In general, a shape-based reconstruction method that recovers dynamic fluorescent yield with a level set method is proposed in this paper. This method is superior to the conventional method that reconstructs only a static distribution of fluorescent yield for the reconstruction of dynamic FMT, because it is capable of resolving the fluorescent yields at each projection angle even if the fluorophore concentrations vary rapidly. Moreover, the target metabolic curves are not restricted by specific models.

## Abbreviations

FMT: fluorescence molecular tomography
FEM: finite element method
ICG: indocyanine green
EMCCD: electron multiplying charge-coupled device
